# A systematic review for pursuing the presence of antibiotic associated enterocolitis caused by methicillin resistant *Staphylococcus aureus*

**DOI:** 10.1186/1471-2334-14-247

**Published:** 2014-05-09

**Authors:** Kentaro Iwata, Asako Doi, Takahiko Fukuchi, Goh Ohji, Yuko Shirota, Tetsuya Sakai, Hiroki Kagawa

**Affiliations:** 1 Division of Infectious Disease, Kobe University Hospital, 7-5-2 Kusunokicho, Chuoku, 650-0017 Kobe, Hyogo, Japan; 2 Division of Infectious Diseases, Kobe City Medical Center General Hospital, 2-1-1 Minamicho, Mintojima, Chuoku, 650-0047 Kobe, Hyogo, Japan; 3 Department of Pediatrics, Himeji Red Cross Hospital, 1-12-1 Shimoteno, 670-0063 Himeji, Hyogo, Japan; 4 Department of Radiology, Kobe University Hospital, 7-5-2 Kusunokicho, Chuoku, 650-0017 Kobe, Hyogo, Japan; 5 Division of Infectious Diseases, Shinko Hospital, 1-4-47 Wakihamacho, Chuoku, 651-0072 Kobe, Hyogo, Japan

**Keywords:** Methicillin-resistant *Staphylococcus aureus*, MRSA, Enterocolitis, Pseudomembranous colitis, *Clostridium difficile*

## Abstract

**Background:**

Although it has received a degree of notoriety as a cause for antibiotic-associated enterocolitis (AAE), the role of methicillin resistant *Staphylococcus aureus* (MRSA) in the pathogenesis of this disease remains enigmatic despite a multitude of efforts, and previous studies have failed to conclude whether MRSA can cause AAE. Numerous cases of AAE caused by MRSA have been reported from Japan; however, due to the fact that these reports were written in the Japanese language and a good portion lacked scientific rigor, many of these reports went unnoticed.

**Methods:**

We conducted a systematic review of pertinent literatures to verify the existence of AAE caused by MRSA. We modified and applied methods in common use today and used a total of 9 criteria to prove the existence of AAE caused by *Klebsiella oxytoca*. MEDLINE/Pubmed, Excerpta Medica Database (EMBASE), the Cochrane Database of Systematic Reviews, and the Japan Medical Abstract Society database were searched for studies published prior to March 2013.

**Results:**

A total of 1,999 articles were retrieved for evaluation. Forty-five case reports/series and 9 basic studies were reviewed in detail. We successfully identified articles reporting AAE with pathological and microscopic findings supporting MRSA as the etiological agent. We also found comparative studies involving the use of healthy subjects, and studies detecting probable toxins. In addition, we found animal models in which enteritis was induced by introducing MRSA from patients. Although we were unable to identify a single study that encompasses all of the defined criteria, we were able to fulfill all 9 elements of the criteria by collectively analyzing multiple studies.

**Conclusions:**

AAE caused by MRSA—although likely to be rarer than previous Japanese literatures have suggested—most likely does exist.

## Background

Although numerous papers have been published to date on methicillin resistant *Staphylococcus aureus* (MRSA), its role in the pathogenesis of antibiotic-associated enterocolitis remains unclear. During the 1950s, *Staphylococcus aureus* was prime suspect in antibiotic-associated pseudomembranous enterocolitis [1]; however, since the identification of *Clostridium difficile* and its toxin as the primary cause of pseudomembranous colitis in the 1970s, *S. aureus* has all but been exonerated from the accusation [2-5]. In more recent years, the majority of cases involving antibiotic-associated enterocolitis (AAE) are considered to be caused by *Clostridium difficile*. In most countries, MRSA as a potential cause of enterocolitis is usually disregarded, if not rarely questioned [6,7].

On the opposite end of the spectrum, a fairly consistent number of MRSA enterocolitis cases have been reported from Japan since the late 1980s [8-10]. Unfortunately, most of these literatures were published in the Japanese language and therefore were rarely acknowledged by the greater international scientific community. The end result was the fueling of a misconception for AAE which lingers to this day [11].

The reports on the so-called MRSA enterocolitis in Japan may have misrepresented *C. difficile* infection (CDI), since routine stool cultures often fail to identify *C. difficile*. This is compounded by the fact that the mere presence of MRSA in stool does not prove its pathogenicity. In addition, oral metronidazole had not been approved for treating CDI in Japan until 2012 [12]. In previous years, oral vancomycin remained the only treatment method available, and many physicians could have mistakenly prescribed a treatment regimen for “MRSA enterocolitis” when in fact they were treating CDI.

In order to elucidate the true causality of MRSA for antibiotic-associated enterocolitis, and bridge the divide on the understanding of AAE among different nations, we conducted a systematic review on MRSA enterocolitis associated with the use of antibiotics.

## Methods

### Search strategy and selection criteria

We systematically searched MEDLINE/PubMed, Embase, the Cochrane Database of Systematic Reviews, and the Japan Medical Abstract Society database for articles available on the subject prior to March 2013. The Japan Medical Abstract Society database is the largest database for medical articles in Japan and contains over 5,000 journals. The repository also consists of over 7.5 million published articles and abstracts of scientific meetings dating back to 1983, written mainly in the Japanese language [13]. We decided to include this database since most reports on MRSA enterocolitis are in fact from Japan but written in the Japanese language and thus may hinder their chances of inclusion in other noteworthy databases. For Pubmed, Embase, and Cochrane, the keywords used for searches included the following: ‘methicillin’/exp OR methicillin AND resistant AND (‘staphylococcus’/exp OR staphylococcus) AND aureus OR ‘mrsa’/exp OR mrsa AND (‘colitis’/exp OR colitis OR ‘enteritis’/exp OR enteritis OR ‘enterocolitis’/exp OR enterocolitis OR ‘diarrhea’/exp OR diarrhea). For the Japan Medical Abstract Society database, “methicillin resistant Staphylococcus aureus,” or “MRSA” AND “choen” (meaning colitis in Japanese) were used for the search. Retrieved reports were also manually screened for further potentially relevant full articles. We included studies written in languages other than English, but excluded studies presented solely as abstracts for scientific conferences.

### Data extraction and evaluation

We included original articles in which antibiotic-associated MRSA enterocolitis was described. To supplement the clinical studies, we also sought basic scientific studies such as those using animals to strengthen the argument for pathogenesis of AAE caused by MRSA, using same search criteria. Eligible reports were independently reviewed for evaluation by two authors (K. I. and D. A.), who are both infectious diseases specialists, certified by the Japanese Association for Infectious Diseases. K. I. is also certified by the American Board of Internal Medicine on both Internal Medicine and Infectious Diseases. Any disputes were resolved by discussion between these two parties.

In general, Koch’s postulates is used for proving a given organism to be the pathogen of any given infectious disease; i.e., 1) the organism must be present in cases of the disease, 2) the organism can be isolated from the diseased host and grown in pure culture, 3) the organism from the pure culture must cause the disease when inoculated into a healthy subject, and 4) the organism must be re-isolated from the diseased subject and shown to be the same organism as the originally inoculated organism [14]. Since MRSA can be found in the stool of healthy subjects, we avoided simply applying Koch’s postulates to prove causality. Likewise, we did not merely apply guidelines proposed for the diagnosis of acute bacterial diarrheal illnesses, since these were developed for pathogens that readily cause outbreaks [14]. Rather, we modified and applied the methods used by Högenauer *et al*. for proving the causality of *Klebsiella oxytoca* as a cause of AAE [15]; i.e., 1) exposure to antibiotics prior to the onset of diarrhea, 2) endoscopic findings consistent with AAE, 3) Gram staining or other microscopic examination of stool or colon consistent with AAE caused by Gram positive cocci in cluster, 4) stool samples demonstrating the presence of MRSA, while excluding other pathogens such as *Clostridium difficile,* 5) comparison with healthy subjects (modified to accommodate comparisons with patients with CDI), 6) toxin assay to identify potential/plausible toxin(s), which is/are found in case but not in control, 7) animal models, with histologic, microbiological evidence of AAE caused by MRSA. We also added that; 8) patient’s condition improves after the use of anti-MRSA agents [7], since it will strengthen the likelihood of MRSA being the causative agent, unlike AAE caused by *K. oxytoca,* which does not require specific treatment if the causative antibiotic was discontinued. We also excluded cases of toxic shock syndrome (TSS) and food poisoning (Criterion 9). Although diarrhea is often observed in TSS caused by *Staphylococcus aureus*, we considered TSS a different entity from antibiotic-associated enterocolitis. Similarly, we tried to distinguish reports of diarrhea, suspected to be caused by food poisoning, which is a well-known self-limiting disease caused by *S. aureus* but not to be confused with antibiotic-associated enterocolitis—which often requires specific antibiotic treatment.

We considered the use of selective, anaerobic stool cultures for *C. difficile* as the ideal method to exclude CDI since it is the most sensitive [16,17]. Absence of *C. difficile* by conventional stool culture was not considered sufficient to exclude CDI, since it has a low sensitivity. Although the *C. difficile* toxin assay is frequently used for the diagnosis of CDI in clinical settings, it lacks the sensitivity to exclude CDI [16]. Therefore, we considered the use of toxin assays as “moderate” evidence for exclusion of CDI, and reports describing the use of these assays were discussed accordingly. The stool glutamate dehydrogenase assay (GDH), a commonly used assay for the diagnosis of CDI, has relatively high negative predictive value [18,19], and we considered that negative GDH results could serve as moderate evidence for exclusion of CDI. Until the late 2000’s, stool assays for both toxin A (TcdA) and B (TcdB) were not available in Japan, and the presence of TcdA only had been examined [20]. Since about 6% of *C. difficile* are non-TcdA producing/TcdB producing (A-/B+), this was also taken into consideration [20].

Conventional stool cultures often fail to identify *C. difficile* and exposure to antibiotics may leave MRSA as the only identifiable pathogen. Therefore, we excluded studies merely finding MRSA in stool culture without specifically attempting to exclude CDI.

## Results

### Literature review

**Figure 1:**
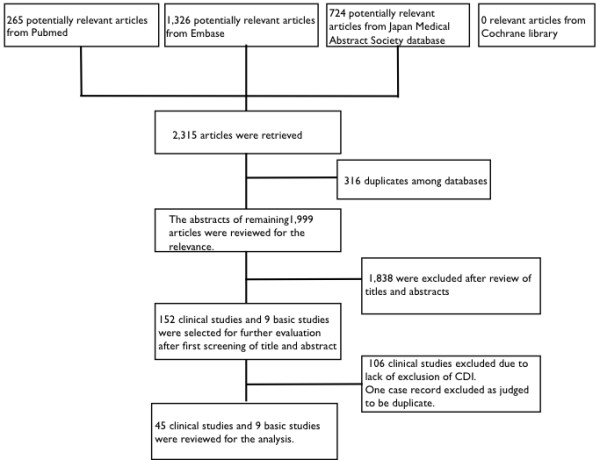
Study selection.

The electronic search identified 2,315 titles (265 from Pubmed, 1,326 from Embase, 0 from the Cochrane library, and 724 from the Japan Medical Abstract Society database). All titles and abstracts were granted full review (Figure 1).

### Evaluation of included cases

**Figure 2:**
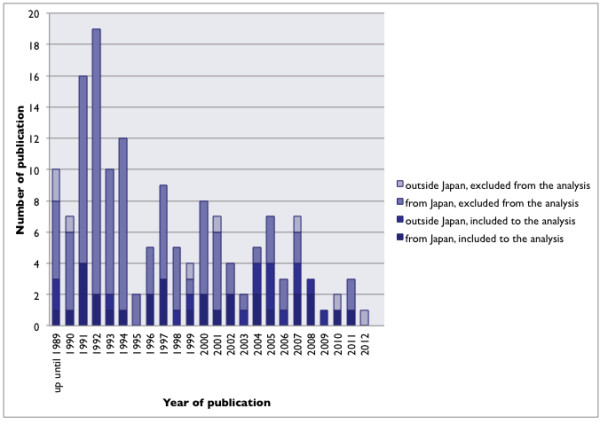
“Studies fulfilling inclusion criteria” and “studies not fulfilling inclusion criteria”.

**Figure 3:**
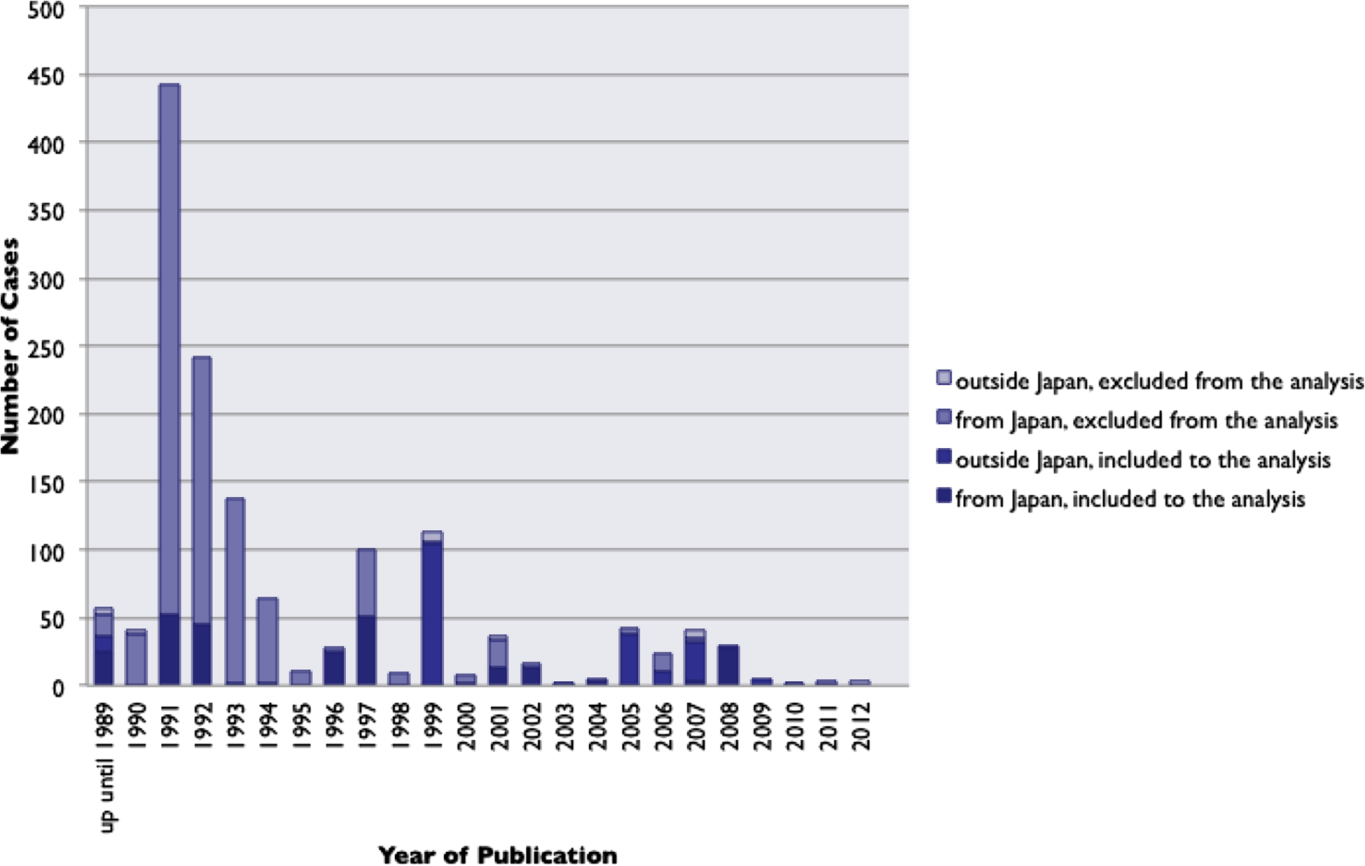
The number of cases presented in published studies by year, both from “studies fulfilling inclusion criteria” and “studies not fulfilling inclusion criteria”.

A total of 152 case reports or case series on a combined total of 1,463 patients were deemed relevant as a result of the initial literature search. The number of articles reporting MRSA enterocolitis has declined over the years (Figure 2), and with that the number of reported cases (Figure 3). Among the selected articles, 31 articles were written in English, one was in Korean, and the remaining 120 articles were written in Japanese. One-hundred-twenty-four articles were from Japan (124/152, 81.6%). Within the 152 articles reviewed, 106 (106/152, 69.1%) were excluded on the basis that they simply diagnosed MRSA enterocolitis based on stool cultures, without making reference to any attempts to exclude CDI. One article written in Japanese [21] was judged to be a duplicate of another case report published in English [22] and therefore the former was removed from the analysis. The remaining 45 articles were reviewed for the pathogenicity of AAE caused by MRSA (Figure 1, and Table 1).

**Table 1:** Summary of Case Records and Case Series on Antibiotic Associated Enterocolitis considered to be caused by MRSA

**Reference**	**Year**	**Country**	**No. of patients**	**Prior exposure to antibiotics**	**Endoscopic findings c/w AAE**	**Gram staining or other microscopic findings**	**Presence of MRSA while excluding CDI**	**Comparison group**	**Toxin assay**	**Animal model**	**Response to the treatment**	**Judged to be different from TSS or food poisoning**
Hori et al. [8].	1989	Japan	154	Some^c^	No	No	Some^d^	No	Yes (SEC, and TSST-1)	No	Yes	Some TSSs
McDonald et al. [23].	1982	Australia	10	Yes	Yes in 1 pt	Yes	Yes by CCFA cx and TA	No	No	No	Yes	Yes
Christie et al. [24].	1988	USA	1	No	No	No	Yes by TA^a^	Yes	Yes^b^	No	Yes	Yes
Sakamoto et al. [25].	1990	Japan	1	Yes	Yes	No	Yes by TA	No	Negative TSST-1	No	Yes	Yes
Matsuo et al. [26].^e^	1991	Japan	34	Yes in 28	4 patients with autopsy findings	No	Yes by CCFA cx.	No	No	No	Yes	No
Nakashio et al. [27].	1991	Japan	1	No	No	No	Yes^d^	No	Yes (SEC and TSST-1)	No	No	Probably TSS^f^
Takeshima et al. [28].	1991	Japan	16	Yes	No	No	Yes by CCMA cx.	No	No	No	No	Yes
Ueda et al. [29].	1991	Japan	2	Yes	No	No	Yes by GDH and cx of unknown methods.	No	Yes (SEA and TSST-1)	No	Yes	UC
Hanatani et al. [30].	1992	Japan	1	Yes	No	Yes	Yes by TA	No	Yes (TSST-1 coding gene)	No	Yes	Yes
Inamatsu et al. [31].	1992	Japan	32	Yes	No	No	Yes by TA and cx of unknown methods.	No	Yes (SE and TSST-1)	No	Yes	One pt might have had TSS
Masuda et al. [32].	1993	Japan	1	Yes	No	Yes	Yes by GDH	No	No	No	Yes	Yes
Taylor et al. [33].	1993	UK	1	Yes	No	No	Yes by TA and cx of unknown methods.	No	Yes (SEA)	No	Yes	Yes
Takatera et al. [34].	1994	Japan	2	Yes	No	No	Yes by GDH	No	No	No	Yes	Yes
Kuramoto et al. [35].	1996	Japan	16	Yes	No	No	Yes by TA	Yes	No	No	Yes	Yes
Watanabe et al. [36].	1996	Japan	9	Yes	No	No	Yes by GDH and cx of unknown methods.	Yes	No	No	Yes	Yes
Konishi et al. [37].	1997	Japan	31	Probably^g^	No	No	Yes by cx of unknown methods.	No	No	No	Yes	Yes
Sakai et al. [38].	1997	Japan	19	Yes	No	No	Yes by GDH and cx of unknown methods.	Compared with CDI	No	No	No	Yes
Yoshida et al. [39].	1997	Japan	1	Yes	Autopsy findings c/w multiple ulcers with abscess. No pseudomembrane formation.	Yes	Yes by unknown methods.	No	Yes (SEC)	No	No	Yes
Schiller et al. [40].	1998	USA	1	Yes	No	Yes	Yes by TA	No	No	No	Yes with ineffectiveness of metronidazole.	Yes
Kimata et al. [22].	1999	Japan	1	No	No	No	Yes by GDH	No	Yes (SEC, TSST-1, and protease B)	No	Yes	Yes
Gravet et al. [41].	1999	France	104	Yes	No	No	Yes by CCFA and TA	Yes^h^	Yes (SEA, SED, LukE-LukD^i^)	No	Yes	Yes
Terada et al. [42].	2000	Japan	1	No	No	No	Yes by TA	No	Yes (TSST-1)	No	Yes	Yes
Watanabe et al^j^[43].	2001	Japan	13	Yes	No	No	Yes by GDH and cx of unknown methods.	No	Yes (SEA, SEC and TSST-1)	No	No	Yes
Yanagi et al. [44].	2002	Japan	1	Yes	No	No	Yes by GDH	No	Yes (SEC and TSST-1)	No	Yes	No
Igami et al. [45].	2002	Japan	13	Yes	No	No	Yes by GDH	No	No	No	Yes	Yes
Lee et al. [46].	2003	S. Korea	1	Yes	Yes	Yes	Yes by TA	No	No	No	Yes	Yes
Fujita et al. [47].	2004	Japan	1	Yes	Autopsy findings c/w PC with cocci	Yes	Yes by GDH	No	No	No	No	No^k^
Yoshida et al. [48].	2004	Japan	1	Yes	Yes	No	Yes by TA	No	No	No	No	Probably necrotizing enterocolitis with Fournier gangrene.
Froberg et al. [49].	2004	USA	1	Yes	Autopsy findings c/w PC	Yes. Also, PCR of pseudomembrane was positive for MRSA.	Positive TA	No	No	No	No	Yes
Rhee et al. [50].	2004	USA	1	Yes	No	No	Yes by TA	No	No	No	Yes (and no response to metronidazole)	Yes
Nishizawa et al. [51].	2005	Japan	1	Yes	No	No	Yes by GDH	No	No	No	Yes	No
Ackermann et al^l^[52].	2005	Germany	25	Yes	No	No	Yes by CCFA cx and TA.	Compared with CDI.	Yes (SEA, SEB, SEC, and SED)	No	UC	No
McPherson et al. [53].	2005	UK	1	Yes	No	No	Yes by TA	No	No	No	Yes	Yes
Boyce et al. [54].	2005	USA	11	Yes			Yes by CCFA cx	Yes	Yes (SEA, SEB, and SED)	No	Yes	Yes
Asha et al. [55].	2006	UK	10	Yes	No	No	Yes by CCEY cx and TA.	Compared with CDI and C. perfringens infections.	Yes (SEA, SEC, SED and TSST-1)	No	UK	Yes
Kurabayashi et al. [56].	2007	Japan	1	Yes	No	Yes	Yes by GDH	No	No	No	Yes	Yes
Nagao et al. [57].	2007	Japan	2	Yes	No	No	positive TA (A only) in cone case. Negative in the other.	No	No	No	Yes, and no response to metronidazole.	Yes
Flemming et al. [58].	2007	Germany	29	Yes	No	No	Yes by CCFA cx and TA	With CDI.	Yes (SEA, SEB, SEC, SED, and SEE)	No	UC	Yes
Kotler et al. [59].	2007	USA	1	No	Yes	Yes (Gram stain of ileal tissue and transmission electron micrograph)	Yes by TA	No	Yes (SEB, SEC)	No	Yes	Co-existence of TSS and enterocolitis.
Shiraishi et al. [60].	2008	Japan	18	UC	No	No	Yes by TA	With CDI	No	No	Yes	Yes
Tamura et al. [61].	2008	Japan	10	Yes	No	No	Yes by GDH	With CDI	No	No	Yes	Yes
Dalal et al. [62].	2008	USA	2	Yes	No	Yes	Yes by TA	No	No	No	Yes	Yes
Lo et al. [63].	2009	USA	5	Yes	No	No	Yes by TA	No	No	No	Yes	Yes
Fujii et al. [64].	2010	Japan	1	UC	No	No	Yes by TA	No	No	No	Yes	Yes
Kitahata Y et al. [65].	2011	Japan	1	Yes	Yes	No	Yes by GDH	No	No	No	Yes	Yes

*Abbreviations*: *MRSA* methicillin-resistant *Staphylococcus aureus*, *c/w* consistent with, *AAE* antibiotic associated enterocolitis, *CDI Clostridium difficile* infection, *TSS* toxic shock syndrome, *pt* patient, *CCFA* cycloserine-cefoxitin-fructose agar, *cx* culture, *TA* toxin assay, *SEC* staphylococcal enterotoxin C, *TSST-1* toxic shock syndrome toxin-1, *CCMA* cycloserine-cefoxitin-mannitol ager, *GDH* glutamate dehydrogenase assay, *SEA* staphylococcal enterotoxin A, *SE* staphylococcal enterotoxins, *SED* staphylococcal enterotoxin D, *SEB* staphylococcal enterotoxin B, *UC* unclear, *PC* pseudomembranous colitis, *CCEY* cycloserine-cefoxitin egg yolk agar, *SEE* staphylococcal enterotoxin E

a. There was no specification whether *S. aureus* in this article was indeed MRSA. However, the patient was treated with oral vancomycin, suggesting it was.

b. Cytopathic effects (CPE) were seen both via intracellular growth of *S. aureus* and with cell-free supernatant, suggesting the presence of toxin in *S. aureus* from the case. CPEs were not seen both in control *S. aureus* and its supernatant.

c. Among the patients identified, the analysis was done for the use of antibiotics in 30 cases.

d. The article did refer to *C. difficile* but did not specify the methods to identify it.

e. Seventeen out of 34 patients did have concurrent *C. difficile* infection.

f. The patient developed septic shock with multiple organ failure, with identification of MRSA from multiple sites.

g. All cases are postoperative. Although there was no specific reference on the use of antibiotics, most patients in Japan usually receive varieties of antibiotics routinely after surgery, particularly in 1980s and 90s.

h. Randomly selected S. aureus isolates were used for comparison of toxin productions.

i. LukE-LukD are one of staphylococcal leukotoxin family [66].

j. It is possible that some patients in this paper might been described in reference 37 in duplicates.

k. The patient died of shock and organ failure. The blood culture after the death grew MRSA.

l. All isolates of *S. aureus* in this study was oxacillin susceptible.

Among these 45 articles, no single study fulfilled all 9 of the criteria we proposed (Table 2). The maximum number of criteria fulfilled by a single study was 6 criteria (6 studies), and the minimum was 2 (one study). Only 8 studies utilized selective culture media for excluding CDI [23,26,28,41,52,54,55,58] and it should be further noted that one of the articles described AAE due to methicillin-sensitive *S. aureus*[52]. There was no animal study accompanying clinical data. Three studies demonstrated the presence of cocci in pathological specimens, which strongly suggested MRSA as being the cause of enterocolitis [47,49,59]. Froberg *et al.* also detected genes uniquely expressed in MRSA (*mecA*) in the pseudomembrane [49]. Comparison with either stools from healthy subjects, [24,35,36,41,54] or clinical features with CDIs [38,52,55,58,60,61] were made in 11 studies. Eighteen studies provided data on toxin production, such as enterotoxins, toxic shock syndrome toxin-1 (TSST-1), protease B, and leucotoxins (LukE-LukD). In one study, the cytopathic effects (CPE) were observed via both intracellular growth of *S. aureus* and with cell-free supernatant, suggesting the presence of toxin in *S. aureus* in the cases presented. CPEs were not observed in either control *S. aureus* or its supernatant [24].

**Table 2:** Summary of the fulfillment of proposed diagnostic criteria of AAE by MRSA

**Criterion #**	**Criterion 1**	**Criterion 2**	**Criterion 3**	**Criterion 4**	**Criterion 5**	**Criterion 6**	**Criterion 7**	**Criterion 8**	**Criterion 9**
**Criterion**	**Prior exposure to antibiotics**	**Endoscopic findings c/w AAE**	**Gram staining or other microscopic findings**	**Presence of MRSA while excluding CDI**	**Comparison group**	**Toxin assay**	**Animal model**	**Response to the treatment**	**Judged to be different from TSS or food poisoning**
Number of studies fulfilled the criterion	38	10	11	45	11	18	0	34	34

*Abbreviations*: *MRSA* methicillin-resistant *Staphylococcus aureus*, *c/w* consistent with, *AAE* antibiotic associated enterocolitis, *CDI Clostridium difficile* infection, *TSS* toxic shock syndrome.

### Basic studies including studies utilizing animal models

Basic studies including those using animal experiments were also sought for analyses. We were able to retrieve 9 potential articles, which appeared relevant.

The oldest article we could retrieve was by van Prohaska [67]. He introduced *S. aureus*, isolated from patients with pseudomembranous enterocolitis or from food implicated in human food poisoning, along with its enterotoxins to chinchillas and cats and reproduced pseudomembranous colitis, as confirmed by necropsy [67]. Although he was able to demonstrate that *S. aureus* and its enterotoxin could cause pseudomembranous enterocolitis in chinchillas, it is unlikely that MRSA or AAE was the cause. Kawai used a rat model with or without gastrectomy and introduced MRSA obtained from patients with diarrhea (positive SEC, TSST-1) after administering kanamycin and metronidazole. The same study confirmed that MRSA multiplied in the rat intestine but failed to demonstrate the occurrence of enterocolitis [68]. Likewise, one study used a mouse model, and showed that the use of ampicillin improved the colonization of MRSA in the intestines. The same study, however, did not directly examine the pathogenesis of MRSA as the cause of enterocolitis [69]. Similar studies reporting strikingly similar results were conducted by different groups using mice [70], and rats [71]. Arima used a rat model by introducing MRSA from a patient with postoperative MRSA enterocolitis who had been administered kanamycin and metronidazole. MRSA colonized in rat intestines, and after administrating latamoxef (oxacephem antibiotic) and cyclophosphamide, MRSA growth significantly increased, which ended in bacteremia and metastatic infections to the liver, kidneys, and spleen [72]. Again, this paper also failed to confirm the occurrence of enterocolitis induced by antibiotic treatment.

Nakamura *et al*. also reported a study using a mouse model. After injecting cyclophosphamide and inducing an immunocompromised state in mice, the group introduced MRSA from patients with postoperative enterocolitis into the jejunum of the mice. After injecting ampicillin and moxalactam for 5 days, they succeeded in producing severe diarrhea in mice, with histologically confirmed inflammation in the jejunum [73]. Although the experiment was rather artificial and the jejunum is not a typical location for AAE, this study was the first—to our knowledge—to histologically describe AAE caused by MRSA in an animal model. A similar form of regional enteritis was demonstrated by others practicing the same method [74,75].

## Discussion

Staphylococcal enterocolitis was first reported in 1942 in an epidemic in a maternity unit [76]. Staphylococcal enterocolitis in association with the administration of antibiotics was first reported in 1948 [77]. Since then, cases of AAE caused by *S. aureus* continued to be reported [67,78,79] and by the end of the 1950s, *S. aureus* was considered to be one of the major causes of AAE [80].

However, since *Clostridium difficile* and its toxin were found to be associated with antibiotic-associated pseudomembranous colitis [2-5], the significance of *S. aureus* was all but marginalized. John G. Bartlett, one of the world’s most well-known infectious diseases specialists and one of the discoverers of *C. difficile* as the cause of pseudomembranous colitis, stated that, “*Staph. aureus* was implicated as the chief cause of antibiotic-associated pseudomembranous enterocolitis in the 1950s. It is unclear whether this finding represented misdiagnosis of *C. difficile* infection or *Staph. aureus* caused a different disease” [1], implying that AAE by S. aureus might not exist, but instead CDI with positive MRSA cultures could have been misinterpreted as AAE by MRSA. Subsequently, routine stool cultures in the hospital setting were discontinued in the United States [81], and physicians virtually ceased to seek out *S. aureus* as a cause of AAE. Only the *C. difficile* toxin assay became the method for diagnosing AAE [16]. The world’s leading textbooks on both Internal Medicine and Infectious Diseases no longer refer to *S. aureus* as a cause of AAE [82,83]. UpToDate and DynaMed, both popular electronic clinical resources, do not lead to sections related to MRSA enterocolitis by using keywords such as “antibiotic associated diarrhea,” or “staphylococcus aureus” (as of May 23, 2013). The Infectious Diseases Society of America, or IDSA, guidelines published on MRSA also has no mention of enterocolitis associated with this particular pathogen [84].

In contrast, numerous articles on MRSA enterocolitis have been published from Japan since the late 1980’s, as shown in our systematic review. Most were written in the Japanese language and consequently went largely unnoticed overseas. To add to the dilemma, most studies published in Japan failed to exclude CDI from the differential. Many failed to make any attempt at searching for *C. difficile* and some used less sensitive tests, such as GDH assays as exclusion criteria for CDI. A *C. difficile* toxin assay for both CdtA and CdtB was approved in 2007 in Japan [20,85], however, prior to this assays for only CdtA had been used, and many studies claiming to have excluded CDI by toxin assay might have failed to detect CDI producing only CdtB. Furthermore, metronidazole was not approved for the treatment of CDI until 2012 in Japan [12], and oral vancomycin was the only antibiotic used for the treatment of AAE. Consistent with Bartnett’s implication, this may have resulted in confusion, which erroneously lead physicians to believe that they were treating MRSA enterocolitis. The lack of similar reports abroad, as well as the lack of studies with scientific rigor cultivated a sense of skepticism to spread amongst Japanese physicians. To add fuel to this skepticism, articles regarding AAE by MRSA, even from Japan, have declined in recent years.

But does this mean that AAE caused by MRSA simply does not exist, and that the apparent epidemic in Japan can be traced to mere ill-definition?

Our answer, based on this systemic review is both “yes” and “no.” While largely ignored in many countries such as the United States, the current systematic review suggests AAE by MRSA most likely exists. Likewise, the majority of cases reported from Japan were probably not AAE by MRSA, and many of these cases were simply a misdiagnosis of CDI (with occasionally different causative entities such as TSS).

In more recent years, experts in both Europe and the USA have rekindled their interests in *S. aureus* as a potential cause for AAE [62,86-88]. It is also important to note that other pathogens such as *Klebsiella oxytoca* have also been found to cause AAE [15].

Our investigation ultimately failed to show any single study on AAE caused by MRSA matching the scientific rigor embodied in the report published by Högenauer *et al.* for the diagnosis of AAE by *K. oxytoca*, which collected clinical cases, examined cytotoxin produced by *K. oxytoca*, compared stool samples with that from healthy subjects, and even established an *in vivo* animal model [15]. However, our systematic review could fulfill all of the established criteria by collectively analyzing data from multiple studies. We found clinical cases of enterocolitis associated with antibiotic use. Studies demonstrated the existence of MRSA in patients, while excluding CDI with sensitive culture media. There was histopathological confirmation on the presence of Gram positive cocci in lesions with inflammation from patients with AAE, which makes the argument for misinterpretation of CDI very unlikely. Comparisons with stool from healthy subjects were conducted, production of toxins was demonstrated, and animal models were established. Even though MRSA enterocolitis associated with antibiotic use may not be all that common—which is in stark contrast to what Japanese physicians had believed until recently—it probably does exist to some extent.

Our review has several inherent limitations. First, we may not have been able to identify all relevant studies, although the independent selection of studies, including those written in non-English language from the Japan Medical Abstract Society database, by two independent reviewers suggests adequate identification among published literatures. Likewise, we did not search for unpublished literatures, which could have relevance to our study. Second, although we were able to fulfill the criteria for the presence of AAE caused by MRSA collectively, no single study independently did so, which slightly weakens the validity of our hypothesis. Third, even if AAE caused by MRSA truly exists, we were unable to demonstrate its clinical significance, including its incidence, clinical characteristics, risk factors, economic impacts, and its consequence such as mortality. Flemming *et al*. identified MRSA in 29 out of 2,727 stool samples (1.1%) of nosocomial diarrhea [58], implying that AAE caused by MRSA is relatively rare, but not extremely uncommon. However, it is well known that the prevalence of MRSA differs significantly from one country to the other [89], and elucidation of the exact incidence of AAE by MRSA warrants independent investigation in each geographical region or country. These clinically relevant agendas should be evaluated in future studies.

## Conclusions

Even though many cases reported mainly from Japan by themselves failed to verify the existence of Antibiotic Associated Enterocolitis caused by MRSA, our systematic review suggests it does probably exist.

## Competing interests

No external source of funding played a role in the study design, implementation, analysis, manuscript preparation, or decision to submit the manuscript for publication. The authors declare that they have no competing interests.

## Authors’ contributions

KI, DA, GO, and HK participated in the design of the study. KI, DA, TF, YS, and TS conducted data collection and initial analyses. KI and DA conducted in-depth analyses of selected studies. KI prepared the draft of the manuscript. KI and TF prepared the figures. All participated in the revision of the manuscript, read and approved the final manuscript.

## Pre-publication history

The pre-publication history for this paper can be accessed here:

http://www.biomedcentral.com/1471-2334/14/247/prepub
